# A DFT prediction of two-dimensional MB_3_ (M = V, Nb, and Ta) monolayers as excellent anode materials for lithium-ion batteries†

**DOI:** 10.1039/d2ra05111g

**Published:** 2022-10-06

**Authors:** Jiahui Wang, Lina Bai, Xiangru Zhao, Hong Gao, Li Niu

**Affiliations:** Key Laboratory for Photonic and Electronic Bandgap Materials, Ministry of Education, School of Physics and Electronic Engineering, Harbin Normal University Harbin 150025 China shidabailina@163.com

## Abstract

Transition metal borides (MBenes) have recently drawn great attention due to their excellent electrochemical performance as anode materials for lithium-ion batteries (LIBs). Using the structural search code and first-principles calculations, we identify a group of the MB_3_ monolayers (M = V, Nb and Ta) consisting of multiple MB_4_ units interpenetrating with each other. The MB_3_ monolayers with non-chemically active surfaces are stable and have metal-like conduction. As the anode materials for Li-ion storage, the low diffusion barrier, high theoretical capacity, and suitable average open circuit voltage indicate that the MB_3_ monolayers have excellent electrochemical performance, due to the B_3_ chain exposed on the surface improving the Li atoms' direct adsorption. In addition, the adsorbed Li-ions are in an ordered hierarchical arrangement and the substrate structure remains intact at room temperature, which ensures excellent cycling performance. This work provides a novel idea for designing high-performance anode materials for LIBs.

## Abstract 2

The boron-exposed MB_3_ monolayers (M = V, Nb and Ta) formed by interpenetrating MB_4_ units have high Li-ion capacities.

## Graphical abstract

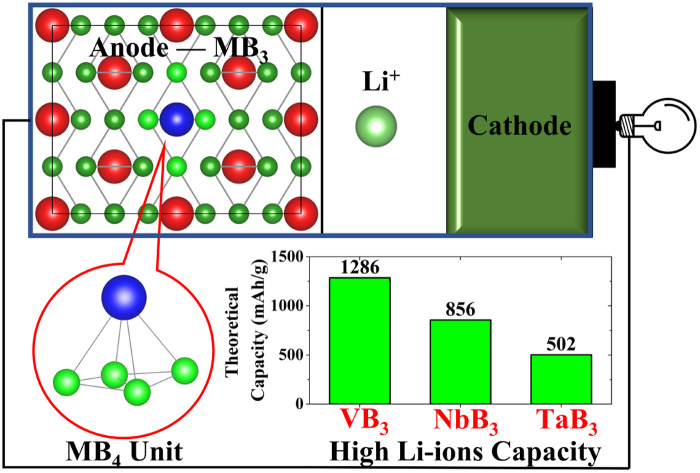

## 1. Introduction

With the rapid development of electronic devices, rechargeable lithium-ion batteries (LIBs) are becoming more and more important in people's life and work,^1^ however, the current commercial electrode materials for LIBs are limited by poor-rate and capacity performance.^2^ Therefore, finding suitable electrode materials to improve performance is a key challenge. Among lots of materials, two-dimensional (2D) materials have been widely applied as the anode materials, due to their high stability, larger surface area, fast charge/discharge rates, and high energy densities. Up to now, a variety of traditional 2D materials for LIBs have been investigated such as graphene,^3,4^ borophene,^5,6^ silicene,^7^ transition metal oxides (TMOs),^8,9^ transition metal dichalcogenides (TMDs).^10–12^

In 2011, with the successfully synthesized transition metal carbides (MXenes) etching by MAX phase,^13^ researchers pay much attention to their electrochemical performance as anode materials.^14–17^ As the representative of the MXenes' family, Ti_3_C_2_, has a high theoretical capacity of 448 mA h g^−1^ for Li-ions^18^ which is higher than graphite. It is regrettable that because the surfaces of MXenes are chemically active, which can absorb many chemical groups in the synthetic process, resulting in the experimental capacity of Ti_3_C_2_ MXene to 123.6 mA h g^−1^ at a rate of 1C,^19^ only 27.5% of theoretical capacity. Zhao *et al.* reported the experimental capacity of Nb_2_C MXene is 342 mA h g^−1^ for Li-ions at a high current density^20^ when O functional groups are increased in a high-temperature synthesis environment, which is higher than 170 mA h g^−1^ at 1C in the past report.^21^ Compared with the theoretical capacity of 813.13 mA h g^−1^, the experimental capacity reached 42%.^22^ It is revealed that the controllable surfaces play a key role which can effectively improve the capacity properties of the anode materials, which have been proven in more reports.^23,24^

The rapid development of structure search has played a great role in the exploration of high-performance anode materials. The exploration of 2D nonmental-rich systems as anode materials of LIBs are concerned widely, such as ScC_2_,^25^ MC_6_ (M = Cu, Ag, Au),^26^ VC_2_,^27^ NiC_3_,^28^ ZrC_2_,^29^ MoC_2_,^30^ and TaC_2_.^31^ On the one hand, Li-ions can directly adsorb on the non-metal elements, which can significantly improve the theoretical capacity. On the other hand, the surfaces of these 2D structures are often chemically inactive, and they will avoid the effects of poorly performing functional groups if they are successfully synthesized experimentally.

A novel family called transition-metal borides (MBenes) has gradually become novel anode materials for LIBs, due to high electronic conductivity and outstanding mechanical properties. In 2017, Guo *et al.* first reported the Mo_2_B_2_ and Fe_2_B_2_ obtained from the MAB phase (M_2_AlB_2_, M represents Mo and Fe) in theory,^32^ which has large theoretical capacities for Li-ions (∼444 and 665 mA h g^−1^). Follow-up studies found that Ti_2_B,^33,34^ Ti_2_B_2_,^35^ V_2_B_2_, Cr_2_B_2_,^36^ Y_2_B_2_,^37^ and Zr_2_B_2_^38^ MBenes also display excellent electrochemical performance. However, the surfaces of the structures listed above are chemically active indicating that these MBenes can be functionalized by chemical groups. Therefore, finding non-chemically active MBenes is an urgent need to improve their performance as electrode materials.

Recently, Li *et al.* reported a novel TiB_3_ monolayer,^39^ whose B atoms are exposed on the surface. A large unit cell area and more adsorption sites provide a theoretical capacity of 1335.4 mA h g^−1^. However, the study of the boron-rich MBenes of other elements as anode materials of LIBs is still lacking. Therefore, we selected group V transition metal elements and designed MB_3_ monolayers (M = V, Nb and Ta) by using the structural prediction method and replacing elements method. Subsequently, their stability, mechanical properties, electronic structures, and electrochemical performance are investigated by using the DFT calculations. Our results show that the MB_3_ monolayers are particularly excellent anode materials for LIBs.

## 2. Computational methods

The CALYPSO code based on the particle swarm optimization (PSO) algorithm was employed to predict structures.^40–42^ The CALYPSO calculation details can be seen in (ESI†). Vienna *Ab initio* Simulation Package (VASP) based on density functional theory (DFT) was employed for structural optimization and electronic structure calculations.^43,44^ The ion–electronic interaction was treated by the project-augmented-wave (PAW) method.^45^ The exchange-correlation functional employed the Perdew–Burke–Ernzerhof (PBE) functional in the generalized gradient approximation (GGA).^46,47^ The kinetic energy cutoff was set to 550 eV in all calculations. The energy and force convergence standards were set as 1 × 10^−5^ eV and 1 × 10^−2^ eV Å^−1^. The spin polarization was turned off during the calculation. The *k*-points meshes in the reciprocal space with the Gamma-center were 2π × 0.25 Å^−1^ and 2π × 0.15 Å^−1^ for structural optimization and electronic structures calculations, respectively. The semi-empirical DFT-D2 method was used to describe the weak van der Waals (vdW) interactions between adatoms and the substrate.^48^

Phonon calculations were performed using the PHONOPY code^49^ in order to demonstrate the dynamic stability, which is based on the density functional perturbation theory (DFPT). Molecular dynamics simulation was performed with the *Ab-initio* Molecular Dynamic (AIMD) simulations. The total time of stimulation was set to 10 ps with a time step of 2 fs (a total of 5000 steps) in the NVT ensemble. The elastic constants were calculated by using the strain–stress method. The energy barriers of diffusion paths were employed by the climbing image nudged elastic band (CI-NEB) method.^50^ The data post-processing used the VASPKIT code.^51^

The cohesive energies of the MB_3_ monolayers can be obtained by the following formula

1 *E*_coh_ = (*E*_MB_3__ − *E*_M_ − 3*E*_B_)/4

where *E*_MB_3__, *E*_M_ and *E*_B_ represent the total energy of the MB_3_ monolayers, an M atom from M-metal phases, and a B atom from borophene monolayer, respectively.

The adsorption energies of Li atoms absorbed on the MB_3_ monolayers are calculated by

2 *E*_ads_ = (*E*_MB_3_Li_*n*__ − *E*_MB_3__ − *nE*_Li_)/*n*

where *E*_MB_3_Li_*n*__ and *E*_Li_ represent the total energy of Li atoms adsorbed on the MB_3_ monolayers and the total energy of a single Li atom obtain by the Li bulk metal. *n* is the layer number of Li atoms.

The theoretical capacities of the MB_3_ monolayers for Li-ions are calculated based on

3 *C* = *nF*/*M*_MB_3__

where *F* is the Faraday constant (26 801 mA h mol^−1^). *M*_MB_3__ represents the molar mass of the MB_3_ monolayers.

In the process of electrochemical reaction, the average open-circuit voltages (OCV) are gained by the following equation^28,31,52^

4 *V* = (*E*_MB_3__ + *nE*_Li_ − *E*_MB_3_Li_*n*__)/*ne*

The in-plane strain (Δ*a* and Δ*b*) and out-of-plane strain (Δ*h*) are calculated by the expressions

5 Δ*a* = (*a*_1_ − *a*) × 100%/*a*

6 Δ*b* = (*b*_1_ − *b*) × 100%/*b*

7 Δ*h* = (*h*_1_ − *h*) × 100%/*h*

where *a*_1_ and *b*_1_ are the lattice constants of the MB_3_Li_*n*_, and *h*_1_ are their thickness. The *a* and *b* are the lattice constants of the MB_3_ monolayers, and *h* are their thickness.

## 3. Results and discussion

### 3.1 Structures and bonding characteristics

**Fig. 1:**
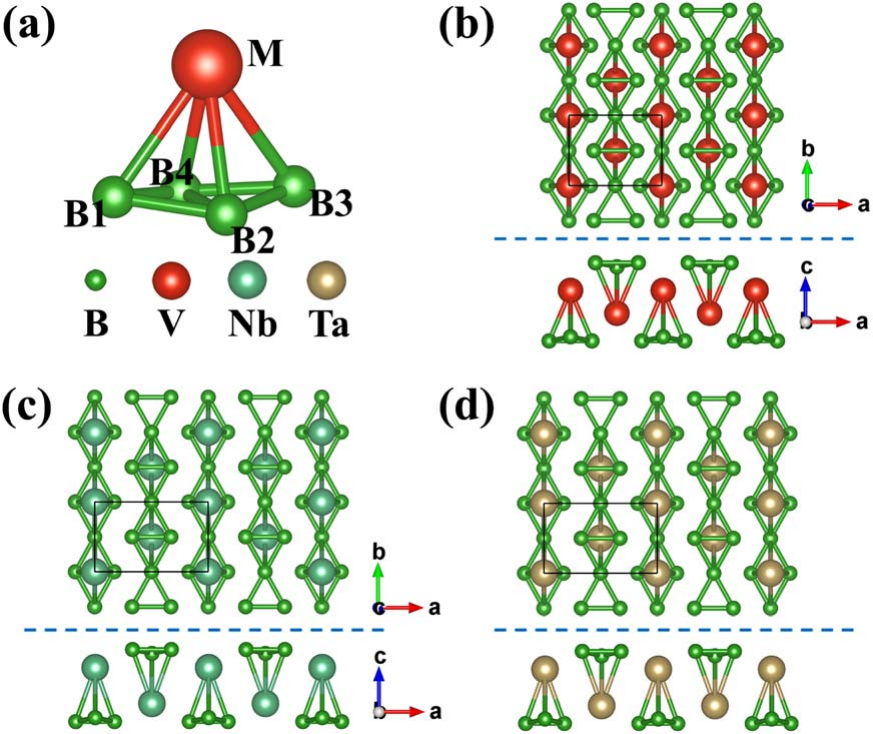
(a) Schematic diagram of MB_4_ structural unit. (b)–(d) Top and side views of the MB_3_ (M = V, Nb, and Ta) monolayers.

The lowest energy configuration of the VB_3_ monolayer is obtained from structural searching and optimization. We use Nb and Ta elements to replace the V element, and obtain reasonable NbB_3_ and TaB_3_ monolayers through structural optimization. These structures belong to *Pmmn* (no. 59) symmetry group and are similar to the TiB_3_ monolayer in the past report.^39^ Each unit cell includes two M atoms and six B atoms, consisting of two MB_4_ units, as shown in Fig. 1. Since the MB_4_ units share the B1 and B3 atoms with each other along the *b*-axis, the stoichiometric ratio of M to B is 1 to 3. The chain B_3_ structure is similar to the B chains in MB_3_ bulk.^53^ These B atoms at the surface form structures similar to C_2_ dimers, which have been proved that such prototype nanocluster structures can stabilize metal clusters^54^ and metal 2D structures,^55^ such as NiC_3_,^28^ TaC_2_,^31^ IrN_2_,^56^ MnB_6_.^57^ The more detailed information of MB_3_ monolayers is listed in Table 1. It can be observed that the addition of Nb and Ta elements increases the lattice constants and bond length of M–M and M–B, and decrease the thickness *h* compared with the VB_3_ monolayer. Furthermore, the NbB_3_ monolayer structure is very similar to the TaB_3_ monolayer.

**Table 1:** Lattice constants (*a*, *b*, unit: Å) and thickness (*h*, unit: Å) of the MB_3_ monolayers. The bond lengths between two atoms (*d*_A–B_, unit: Å)

	*a*	*b*	*h*	*d*_B1–B2_	*d*_B2–B4_	*d*_M–B1_	*d*_M–B4_
VB_3_	3.918	2.960	3.304	1.743	1.809	2.458	2.319
NbB_3_	4.839	2.982	3.081	1.713	1.651	2.606	2.449
TaB_3_	4.834	2.979	3.071	1.714	1.652	2.588	2.452

**Fig. 2:**
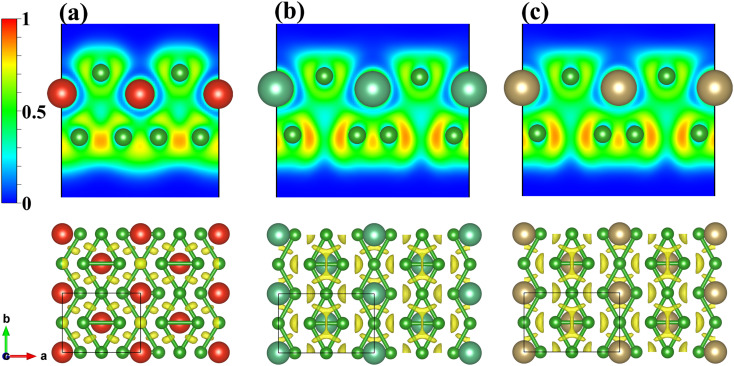
2D ELF maps of the (010) plane (up) and 3D ELF maps (down) for the (a) VB_3_, (b) NbB_3_, and (c) TaB_3_ monolayers. The isosurface for 3D ELF maps is selected as 0.8.

Electron localization function (ELF) maps of the MB_3_ monolayers are plotted to investigate the bonding characteristics, as shown in Fig. 2. ELF values are normalized, where 0, 0.5, and 1 represent extremely low electron densities, completely delocalized electrons, and completely localized electrons, respectively. It can be observed that the electrons are localized between the B atoms which form strong covalent bonds. There are very few electrons around M atoms, indicating the ionic bonds between M and B atoms. It can be observed that the NbB_3_ and TaB_3_ monolayers have more localized electrons between the B2–B4 bonds of the MB_4_ unit compared with the VB_3_ monolayer, indicating a stronger interaction between B2–B4 of the NbB_3_ and TaB_3_ monolayers, as shown in the 3D ELF of Fig. 2.

### 3.2 Stability

The possibility of the MB_3_ monolayers for experimental synthesis can be demonstrated by the cohesive energy, phonon spectrum, and AIMD calculations. Our computed cohesive energies are −0.51 eV for the VB_3_ monolayer, −0.47 eV for the NbB_3_ monolayer, and −0.39 eV for the TaB_3_ monolayer, showing that the MB_3_ monolayers are thematically stable. Their dynamic stability is proved by phonon dispersion curves, as shown in Fig. S2 in ESI.† No negative frequency indicates that the MB_3_ monolayers are dynamically stable. The highest frequencies are up to 28.48 THz (949.99 cm^−1^) for the VB_3_ monolayer, 31.66 THz (1056.06 cm^−1^) for the NbB_3_ monolayer, and 31.64 THz (1055.40 cm^−1^) for the TaB_3_ monolayer at *Γ* point, which represents their outstanding stability. AIMD simulations are also shown in Fig. S2.† The integrity of their structures and their energy oscillations at equilibrium indicate that the MB_3_ monolayers have excellent thermodynamic stability at high temperatures.

Furthermore, the mechanical stability of the MB_3_ monolayers is investigated by elastic constants which are listed in Table 2. There are four independent variables for the rectangular crystal system, which are *c*_11_, *c*_12_, *c*_22_, and *c*_66_, respectively. Their elastic constants satisfy stability criteria: *c*_11_ > 0, *c*_66_ > 0 and (*c*_11_*c*_22_ − *c*_12_^2^) > 0. The corresponding 2D (in-plane) Young's moduli of MB_3_ monolayers are calculated by *Y*_*x*_ = (*c*_11_*c*_22_ − c12^2^)/*c*_22_ and *Y*_*y*_ = (*c*_11_*c*_22_ − c12^2^)/*c*_11_. Their *Y*_*x*_ exceeds 100 N m^−1^ and *Y*_*y*_ exceeds 300 N m^−1^ respectively, indicating their good mechanical stiffness. These stability calculations prove that MB_3_ monolayers can be synthesized as independent nanostructures. It is noted that the NbB_3_ and TaB_3_ monolayers have the larger *Y*_*x*_ and smaller *Y*_*y*_ compared to the VB_3_ monolayer, this result is consistent with the analysis of the bonding characteristics between the B2–B4 for the MB_4_ unit.

**Table 2:** Elastic constants (*c*_11_, *c*_12_, *c*_22_, *c*_66_, unit: N m^−1^) and Young's moduli along *a*- and *b*-axes (*Y*_*x*_ and *Y*_*y*_, unit: N m^−1^) of the MB_3_ monolayers

	*c*_11_	*c*_12_	*c*_22_	*c*_66_	*Y*_*x*_	*Y*_*y*_
VB_3_	115.796	27.621	375.757	171.522	113.766	369.169
NbB_3_	181.063	67.315	334.253	143.037	167.506	309.227
TaB_3_	214.373	75.296	346.031	150.963	197.989	319.584

### 3.3 Electronic structures

**Fig. 3:**
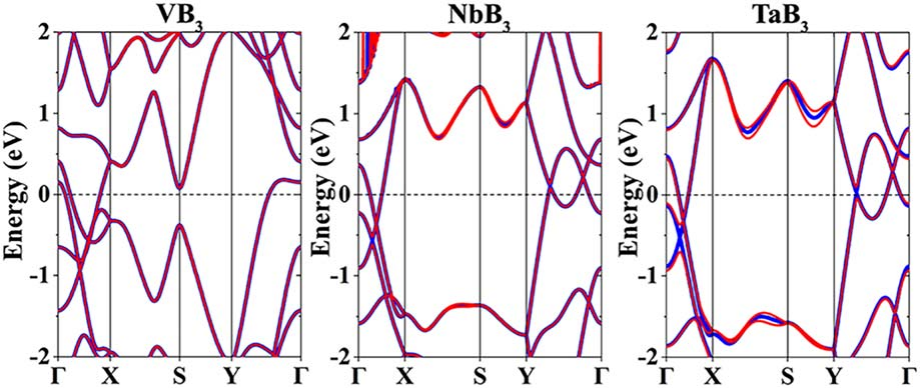
Band structures of the MB_3_ monolayers. The thick blue and thin red lines represent the GGA and GGA + SOC methods, respectively.

The GGA and GGA + SOC methods are both considered to calculate the band structures of the MB_3_ monolayers which are displayed in Fig. 3. They all exhibit metal-like characteristics due to the bands crossing the Fermi level (*E*_F_), indicating high electrical conductivity. The SOC effect cannot change their metallic properties. Therefore, the MB_3_ monolayers have a high electronic conductance. Surprisingly, Dirac cones near the *E*_F_ can be observed in the band structures of the NbB_3_ and TaB_3_ monolayers on *Γ*–S and Y–*Γ* paths in the Brillouin zone (BZ), but not in that of the VB_3_ monolayer.

### 3.4 Adsorption and diffusion of the single Li atom

**Fig. 4:**
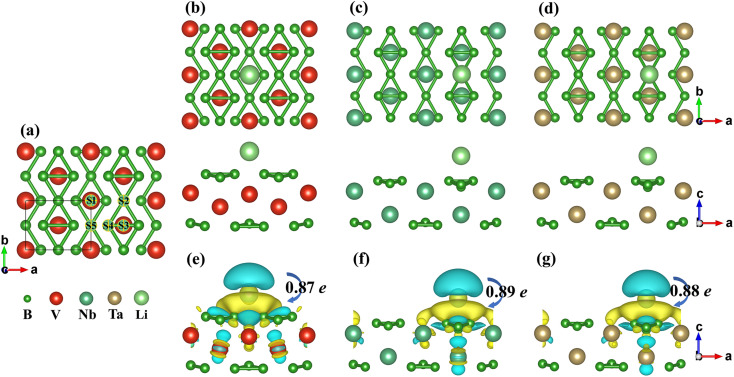
(a) Possible adsorption sites of a single Li atom on the monolayers. (b)–(d) Schematic diagrams of MB_3_Li_0.125_. (e)–(g) The charge density difference maps of MB_3_Li_0.125_. The black numbers represent the amount of charge transferred from the Li atom to the monolayer.

In order to study the adsorption behaviour of a single Li atom on the MB_3_ monolayers, a 2 × 2 × 1 supercell is selected and five atomic positions are considered after full consideration of the symmetry. As shown in Fig. 4(a), the five positions are arranged clockwise from S1 to S5. Their adsorption energies are listed in Table 3 (the value of the adsorption energies under different correction schemes are shown in Table S2–S4†). It should be noted that S4 will move to S3 for the VB_3_ monolayer or S5 for NbB_3_ and TaB_3_ monolayer. Therefore, only four adsorption sites will be considered (S1, S2, S3, and S5) in subsequent calculations. For the VB_3_ monolayer, the adsorption site with the lowest adsorption energy is S1 (on the M atoms). However, S2 (on the B1/B3 atoms) is the most favourable site for NbB_3_ and TaB_3_ monolayers. The most favourable sites of the single Li atom for the MB_3_ monolayers are shown in Fig. 4(b)–(d).

**Table 3:** Adsorption energies (*E*_ads_, unit: eV) of single Li atom adsorbed on the MB_3_ monolayers at different adsorption sites

	S1	S2	S3	S4	S5
VB_3_	−1.418	−0.835	−0.432	−0.432	−0.439
NbB_3_	−0.676	−0.933	−0.487	−0.833	−0.833
TaB_3_	−0.620	−0.963	−0.456	−0.931	−0.931

The charge density difference maps of the favourite Li sites for the MB_3_ monolayers are also shown in Fig. 4(e)–(g). The yellow and blue areas represent the gained and lost electrons, respectively. The transfer of electrons from the Li atom to the MB_3_ surfaces can be observed, indicating that the Li atom forms the ionic bonds with the MB_3_ monolayer. Bader charge analysis can quantitatively analyze the value of the charge transfer from the Li atom to the monolayer, as shown in the black number. This reveals that the Li atom has been completely ionized on the surface, forming the Li-ions.

**Fig. 5:**
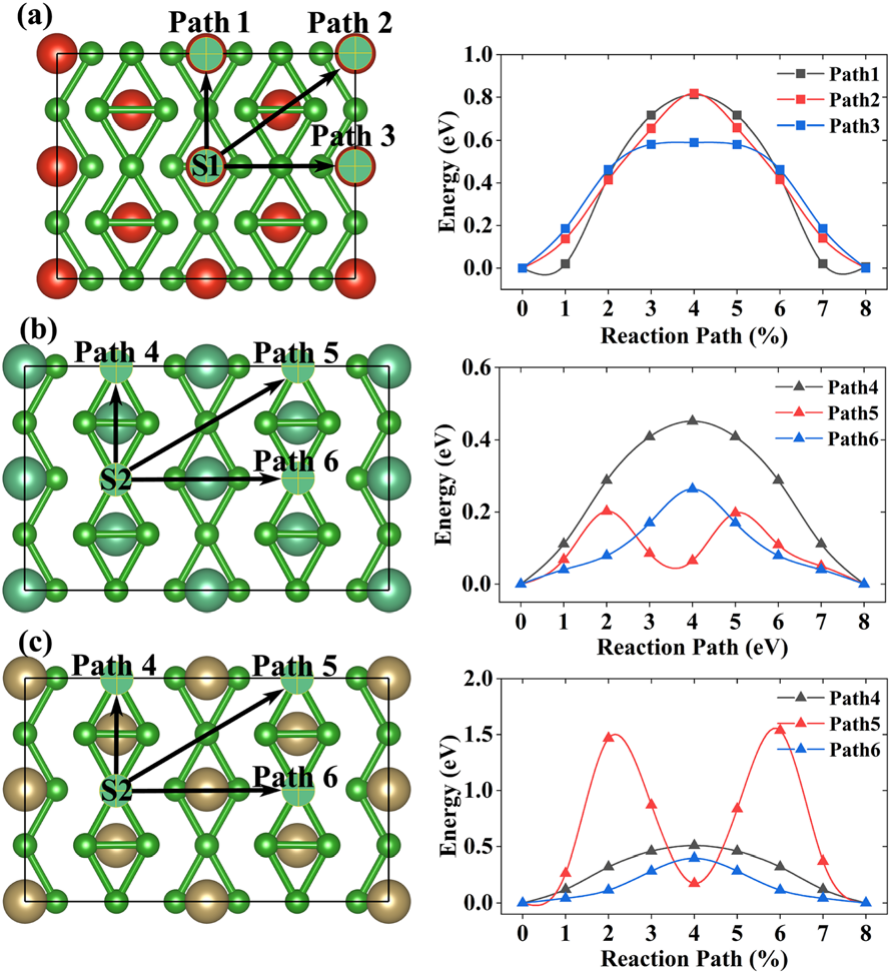
The diffusion path and diffusion barrier of a single Li-ion on the (a)VB_3_, (b)NbB_3_, and (c)TaB_3_ monolayers, respectively.

The diffusion barrier is an important parameter to measure the rate of charge and discharge. High-performance anode materials tend to have low diffusion barriers. According to their adsorption sites with the lowest adsorption energies, six possible diffusion paths are considered, which can be seen in Fig. 5. For the VB_3_ monolayer, the optimal diffusion path is path 3 whose value is 0.590 eV. However, path 5 (0.202 eV) and path 6 (0.399 eV) are the lowest energy diffusion paths for the NbB_3_ monolayer and TaB_3_ monolayer. Compared with other high-performance anode materials for LIB in theory, such as 0.50 eV for NiC_3_,^28^ 0.58 eV for silicon,^58^ 0.60 eV for *χ*_3_ borophene^5^ and 0.78 eV for MoN_2_,^59^ our MB_3_ monolayers have fast charge–discharge rates which could be anode materials for LIBs.

### 3.5 Theoretical capacity and open-circuit voltage

Theoretical capacity and average OCV are important indicators for electrode material, which are related to the number of adatoms and their adsorption energies. In the following discussion, we divide the VB_3_ monolayer into a separate group, and NbB_3_ and TaB_3_ monolayers into another group due to their differences in structural characteristics and the most stable adsorption sites. The Li atoms are stacked on both sides of the monolayer according to the above adsorption sites until these sites exposed to the surface are completely occupied, corresponding to the stoichiometries of MB_3_Li_*n*_ (*n* = 1–4).

**Fig. 6:**
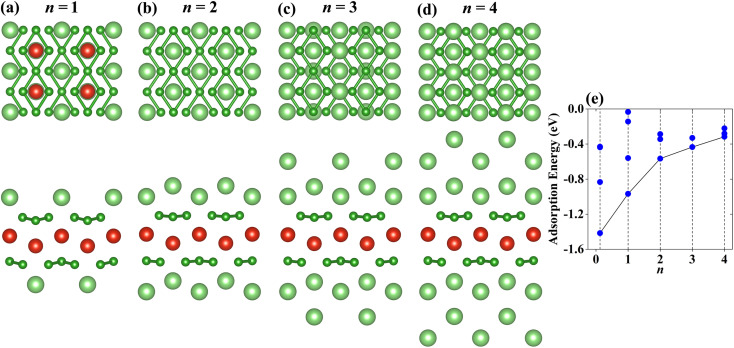
(a)–(d) Top and side views of the most stable adsorption configurations with *n* layer Li-ions adsorbed on the VB_3_ monolayer (*n* = 1, 2, 3 and 4). (e) The adsorption energies of VB_3_Li_*n*_ as a function of Li-ion content. The black lines connect the adsorption energies corresponding to the most stable adsorption configurations.

The configurations of the Li atoms adsorbed on the VB_3_ surfaces with the lowest adsorption energies are shown in Fig. 6(a)–(d). When *n* = 1, the first layer Li atoms prefer to adsorb on S1. With the adatoms increasing, the second Li atomic layer is located at S4. The subsequent third and fourth layers of Li atoms are adsorbed on S5 and S2, respectively. Fig. 6(e) shows the adsorption energies of VB_3_Li_*n*_. With the increase of the Li content, the average adsorption energies increase gradually from −1.416 eV per Li atom (*n* = 0.125) to −0.317 eV per Li atom (*n* = 4) but still remain negative. These results demonstrate that the VB_3_ monolayer can adsorb four-layer Li at least. According to eqn (3), the corresponding theoretical capacity is 1286 mA h g^−1^ which is higher than that of VC_2_ (1073 mA h g^−1^),^27^ V_2_B_2_ (969 mA h g^−1^).^36^ The corresponding average OCV for VB_3_Li_4_ is 0.32 V which is suitable to be an anode material due to the value between 0 and 1 V, indicating the VB_3_ monolayer does not dendrite during lithiation and its capacity is reversible.^52,60^

**Fig. 7:**
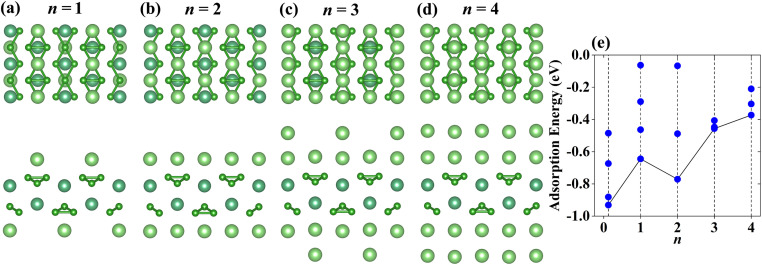
(a)–(d) Top and side views of the most stable adsorption configurations with *n* layer Li-ions adsorbed on the NbB_3_ monolayer (*n* = 1, 2,3 and 4). (e) The adsorption energies of NbB_3_Li_*n*_ as a function of Li-ion content. The black lines connect the adsorption energies corresponding to the most stable adsorption configurations.

**Fig. 8:**
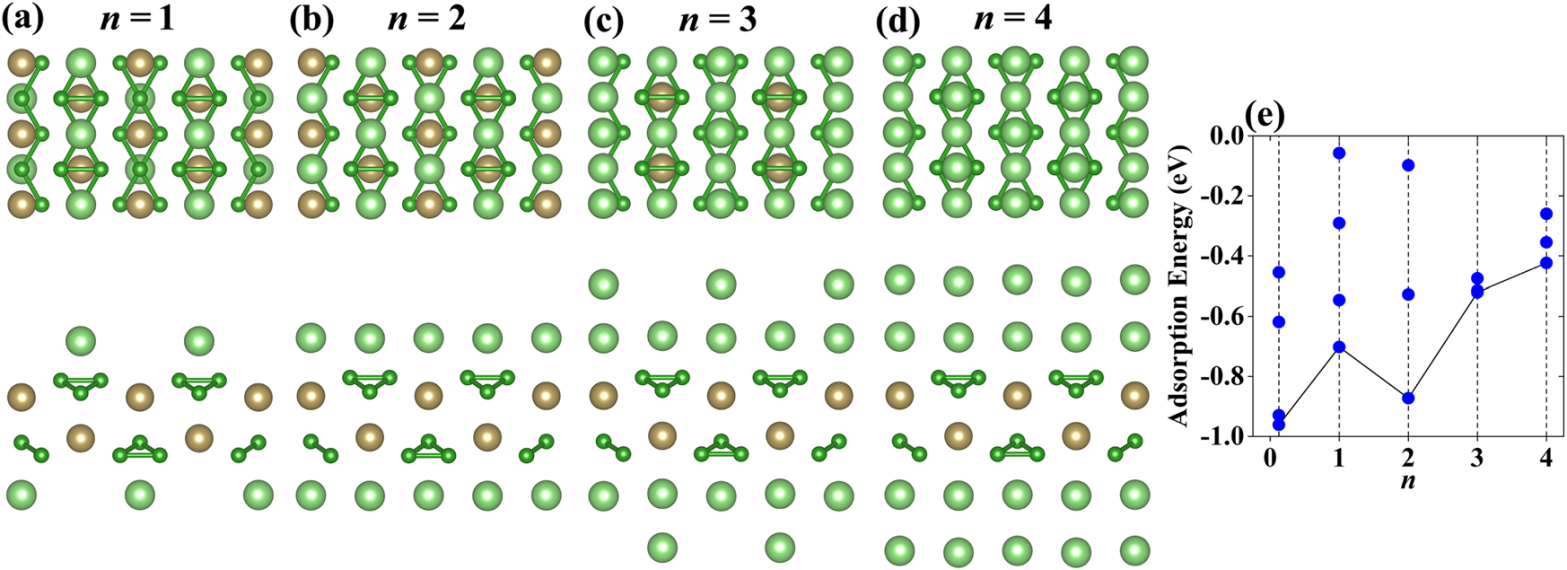
(a)–(d) Top and side views of the most stable adsorption configurations with *n* layer Li-ions adsorbed on the TaB_3_ monolayer (*n* = 1, 2, 3 and 4). (e) The adsorption energies of TaB_3_Li_*n*_ as a function of Li-ion content. The black lines connect the adsorption energies corresponding to the most stable adsorption configurations.

Same to the VB_3_ monolayer, the configurations of the Li atoms adsorbed on NbB_3_ and TaB_3_ monolayers are considered, whose most stable structures can be seen in Fig. 7(a)–(d) and 8(a)–(d). The occupy positions of Li atoms order to S2–S5–S1–S4 from the first layer to the fourth layer. The corresponding adsorption energies can be seen in Fig. 7(e) for NbB_3_Li_*n*_ and Fig. 8(e) for TaB_3_Li_*n*_. With the increase of the layer number of adatom, the adsorption energies increase gradually, except for that of Nb(Ta)B_3_Li_2_ which is lower than that of Nb(Ta)B_3_Li. In addition, the Li atoms adsorbed on the NbB_3_ and TaB_3_ monolayers are neatly arranged, when the value *n* is even, which causes lower adsorption energies for Nb(Ta)B_3_Li_2_ and Nb(Ta)B_3_Li_4_ compared with VB_3_Li_2_ and VB_3_Li_4_ due to the suitable arrangement of Li atoms. The theoretical capacities of the NbB_3_ and TaB_3_ monolayers for Li-ions are 856 mA h g^−1^ and 502 mA h g^−1^, respectively. Compared with Nb and Ta carbides such as 813.12 mA h g^−1^ for Nb_2_C,^22^ 194.36 mA h g^−1^ for Nb_2_CS_2_,^61^ 264 mA h g^−1^ for Ta_2_C and 556 mA h g^−1^ for TaC,^31^ they have excellent theoretical capacities. According to eqn (4), the average OCVs for NbB_3_Li_4_ and TaB_3_Li_4_ are 0.37 V and 0.42 V, indicating that they are potential anode materials for LIBs.

The process of Li adsorption often results in a volume change of anode material, which will affect the stability during the cycling process of electrode material. Therefore, the in-plane strain (Δ*a*: along *a*-direction, Δ*b*: along *b*-direction) and the out-of-plane strain (Δ*h*) during Li adsorption are obtained, which can be seen in Table S6–S8,† according to the eqn (5)–(7). The |Δ*a*| of VB_3_Li_*n*_ stays below 4% and |Δ*b*| remains below 1%. The Δ*h* of the VB_3_Li_*n*_ monolayer increases to a maximum of 3.608% when *n* = 1, and decreases gradually to 0.022% when *n* = 4. However, the values of |Δ*a*| and |Δ*b*| for NbB_3_Li_*n*_ and TaB_3_Li_*n*_ are all below 2%. The maximum values Δ*h* of Nb(Ta)B_3_Li_*n*_ are 4.737% (NbB_3_Li_2_) and 5.607% (TaB_3_Li_3_), respectively. Compared with graphite (Δ*a* ∼ 12%),^62^ Ti_2_CSSe (Δ*a* ∼ 4.10%, Δ*h* ∼ −2.22%), Ti_2_CSO (Δ*h* ∼ 6.75%)^63^ and MC_6_ (Δ*a* ∼ 6.30%),^23^ the volume change of the MB_3_ monolayers is small enough to be excellent anode materials.

The AIMD simulations are employed to verify the thermodynamical stability when MB_3_ monolayers adsorb Li atoms at 300 K, which can be seen in Fig. S3.† Compared with the highly deformed anode materials during the process of lithiation at certain temperatures, such as NiC_3_,^28^ and hydrogenated graphene-like borophene,^64^ the structures of the MB_3_ monolayers are undestroyed by Li-ions. Combined with the volume change of the Li adsorption process, it can clearly see that the MB_3_ monolayer will not cause safety problems when working as an anode material due to volume change (Table 4).

**Table 4:** Comparison of theoretical capacities (*C*_M_, unit: mA h g^−1^) and diffusion barriers (DB, unit: eV) for other anode materials of LIBs

	*C*_M_	DB	Ref.
VB_3_	1286	0.590	This work
V_2_B_2_	969	0.22	36
α-VC_2_	1073	0.52	27
NbB_3_	856	0.202	This work
Nb_2_C	813	0.03	22
Nb_2_CS_2_	194.36	0.23	61
TaB_3_	502	0.399	This work
Ta_2_C	264	0.21	31
TaC	556	0.25	31
MoN_2_	432	0.78	59

## Conclusion

In conclusion, we design boron-rich MB_3_ monolayers (M = V, Nb and Ta) and explore their electrochemical performance as anode materials for LIBs, using the structural prediction method and first-principles calculations. The MB_3_ monolayers are stable which is verified by cohesive energies, phonon dispersions, molecular dynamics simulations and elastic constants, respectively. The high Young's moduli of MB_3_ monolayers mean they have high stiff enough. Electronic structure calculations show that the MB_3_ monolayers have metal-like conduction. Furthermore, the low diffusion barriers and suitable average open-circuit voltages suggest that they could be promising anode materials for LIBs. Most importantly, the theoretical capacities are 1286 mA h g^−1^ (VB_3_Li_4_), 856 mA h g^−1^ (NbB_3_Li_4_) and 502 mA h g^−1^ (TaB_3_Li_4_) which are higher than a lot of 2D anode materials for LIBs. Our work has enriched the study of MBenes as anode materials and provided certain guidance for future theoretical and experimental studies.

## Conflicts of interest

There are no conflicts to declare.

## Supplementary Material

RA-012-D2RA05111G-s001
