# Supplementary material for: A DFT prediction of two-dimensional MB3 (M = V, Nb, and Ta) monolayers as excellent anode materials for lithium-ion batteries
Source: RSC Adv. 2022 Oct 6;12(44):28525–32. doi: 10.1039/d2ra05111g (PMC9535470; doi:10.1039/d2ra05111g)
Supplement: RA-012-D2RA05111G-s001 [file RA-012-D2RA05111G-s001.pdf]

Electronic Supplementary Material (ESI) for RSC Advances.
This journal is © The Royal Society of Chemistry 2022

Electronic Supplementary Information for

A DFT prediction of the two-dimensional MB_3 (M = V, Nb, and Ta) monolayers as excellent anode materials for lithium-ion batteries

Jiahui Wang, Lina Bai,[*] Xiangru Zhao, Hong Gao, Li Niu

Key Laboratory for Photonic and Electronic Bandgap Materials, Ministry of Education, School of Physics and Electronic Engineering, Harbin Normal University, Harbin 150025, China

[*] Corresponding author: Lina Bai; Email: shidabailina@163.com

Computational Details of CALYPSO

The particle swarm optimization (PSO) method within the evolutionary algorithm as implemented in the CALYPSO code was employed to find the low-energy structures of the VB_3 monolayers. The ratio of V atoms to B atoms is 1 to 3. Each generation produces 20 structures for a total of 30 generations. In most cases, structural searching simulations for each calculation were stopped after generating 500 structures. The vacuum layer was set above 20 Å to ensure that the searched structure was free of layer interactions. The local optimization of the structure is divided into three steps, and the accuracy increases gradually to ensure that each generated structure can be fully optimized.

Table S1 The cohesive energies of the MB_3 monolayers by different vdW corrections.

	Cohesive Energy (eV/atom)		
	VB_3	NbB_3	TaB_3
no vdW	− 0.65	− 0.66	− 0.65
D2	− 0.51	− 0.47	− 0.39
D3	− 0.63	− 0.62	− 0.58
D3-BJ	− 0.63	− 0.61	− 0.61

Table S2 The adsorption energies of $MB_3Li_{0.5}$ under GGA-PBE without vdW correction.

	Adsorption Energy (eV)				
	S1	S2	S3	S4	S5
VB_3	**– 0.807**	– 0.366	– 0.048	– 0.048(S3)	– 0.108
NbB_3	– 0.443	**– 0.634**	– 0.307	– 0.619(S5)	– 0.619
TaB_3	– 0.319	**– 0.561**	– 0.205	– 0.527(S5)	– 0.527

Table S3 The adsorption energies of $MB_3Li_{0.5}$ under GGA-PBE with D3 correction.

	Adsorption Energy (eV)				
	S1	S2	S3	S4	S5
VB_3	**– 1.146**	– 0.687	– 0.355	– 0.355(S3)	– 0.417
NbB_3	– 0.601	– 0.744	– 0.417	– 0.767(S5)	**– 0.767**
TaB_3	– 0.455	– 0.662	– 0.249	– 0.670(S5)	**– 0.670**

Table S4 The adsorption energies of $MB_3Li_{0.5}$ under GGA-PBE with D3-BJ correction.

	Adsorption Energy (eV)				
	S1	S2	S3	S4	S5
VB_3	**– 1.114**	– 0.624	– 0.292	– 0.292(S3)	– 0.343
NbB_3	– 0.583	**– 0.743**	– 0.389	– 0.737(S5)	– 0.737
TaB_3	– 0.436	**– 0.668**	– 0.287	– 0.636(S5)	– 0.636

Table S5 The adsorption energies (E_{ads}, unit: eV) of MB_3Li_n (n = 1, 2, 3 and 4).

	E_{ads}		
n	VB_3Li_n	NbB_3Li_n	TaB_3Li_n
1	– 0.968	– 0.645	– 0.702
2	– 0.566	– 0.771	– 0.873

3	– 0.434	– 0.457	– 0.521
4	– 0.317	– 0.373	– 0.423

Table S6 The lattice constants (a, b, unit: Å), in-plane strain (Δa and Δb), layer thickness (h, unit: Å) and out-of-plane strain (Δh) for VB_3Li_n (n = 1, 2, 3 and 4).

	VB_3Li_n					
n	a	Δa	b	Δb	h	Δh
1	7.553	– 3.620 %	5.980	– 1.013 %	3.423	3.608 %
2	7.699	– 1.765 %	5.960	0.679 %	3.375	2.155 %
3	7.658	– 2.228 %	5.964	0.751 %	3.362	1.755 %
4	7.680	– 2.008 %	5.932	0.022 %	3.369	1.961 %

Table S7 The lattice constants (a, b, unit: Å), in-plane strain (Δa and Δb), layer thickness (h, unit: Å) and out-of-plane strain (Δh) for NbB_3Li_n (n = 1, 2, 3 and 4).

	NbB_3Li_n					
n	a	Δa	b	Δb	h	Δh
1	9.847	1.734 %	5.911	– 0.903 %	3.114	1.051 %
2	9.803	1.288 %	5.923	– 0.709 %	3.228	4.737 %
3	9.797	1.222 %	5.929	– 0.598 %	3.168	2.790 %
4	9.803	1.286 %	5.918	– 0.788 %	3.187	3.413 %

Table S8 The lattice constants (a, b, unit: Å), in-plane strain (Δa and Δb), layer thickness (h, unit: Å) and out-of-plane strain (Δh) for TaB_3Li_n (n = 1, 2, 3 and 4).

	TaB_3Li_n					
n	a	Δa	b	Δb	h	Δh
1	9.816	1.537 %	5.874	– 1.422 %	3.112	1.348 %
2	9.751	0.867 %	5.885	– 1.230 %	3.243	5.607 %
3	9.746	0.816 %	5.892	– 1.109 %	3.186	3.758 %
4	9.761	0.968 %	5.885	– 1.233 %	3.186	3.758 %

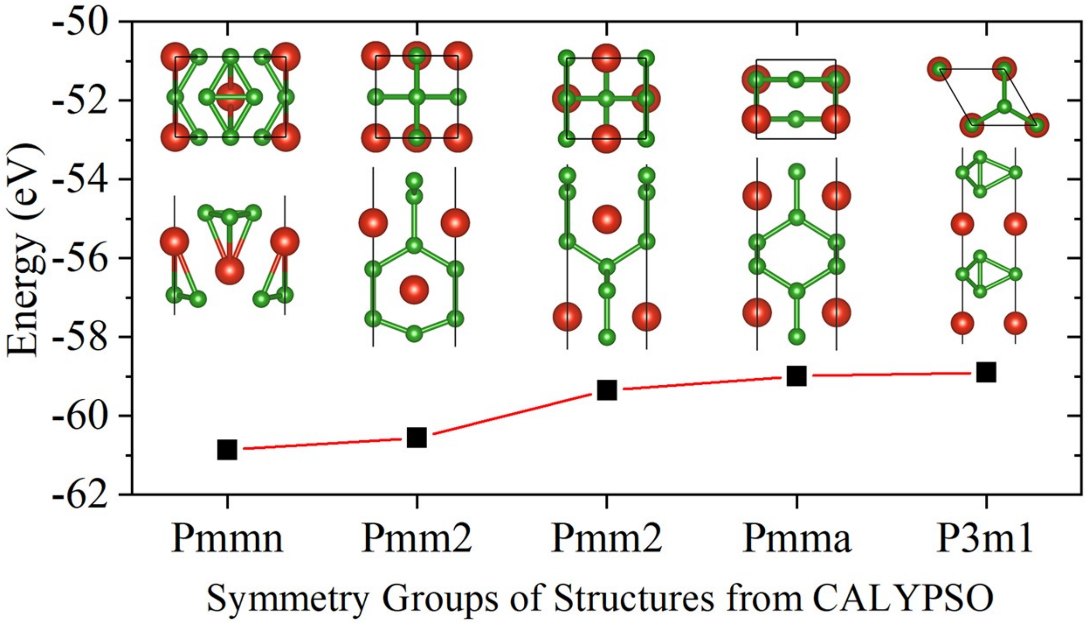

Fig. S1 The low-energy structures obtained by CALYPSO and the energies calculated by VASP. The upper and lower structural diagrams represent the top and side views of the corresponding structures, respectively.

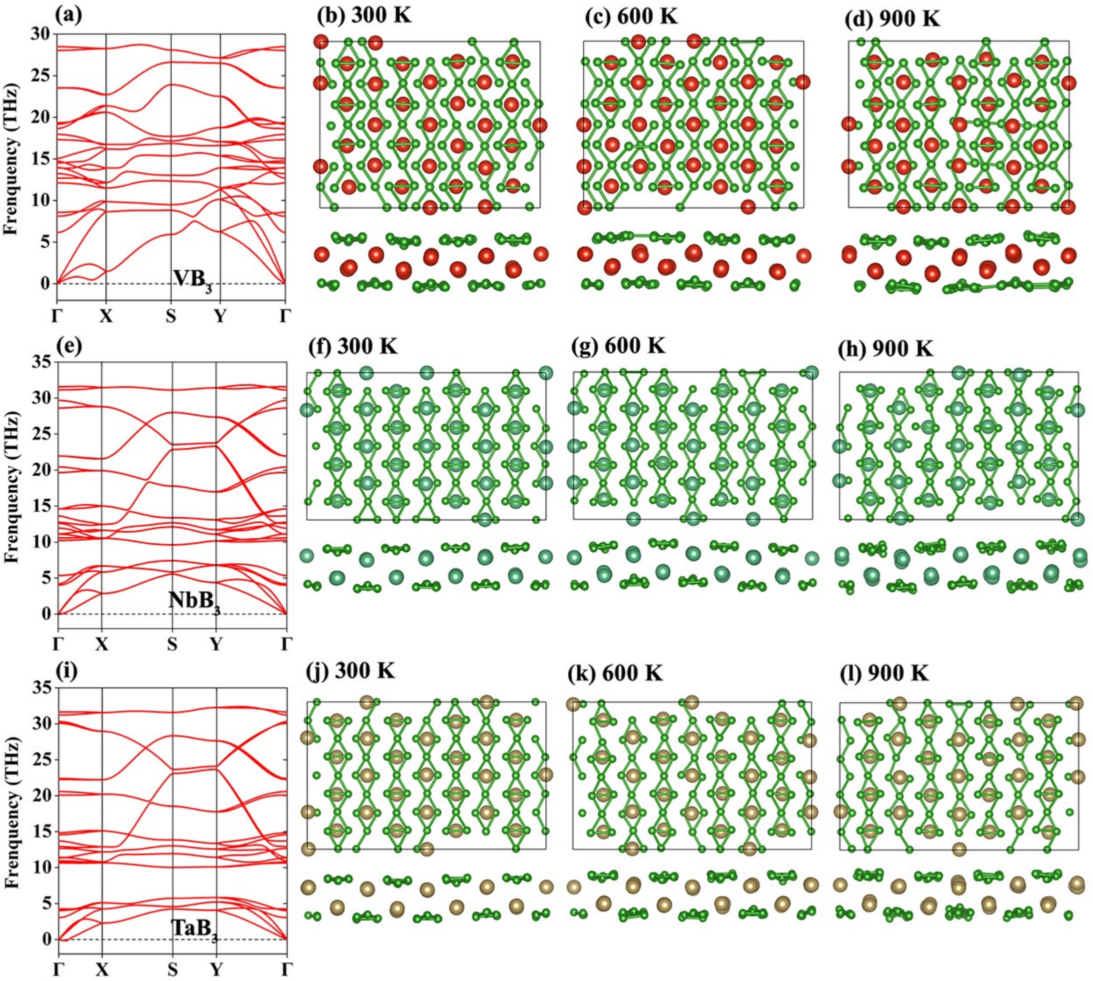

Fig. S2 The phonon dispersion curves and AIMD snapshots of the VB_3, NbB_3 and TaB_3 monolayers.

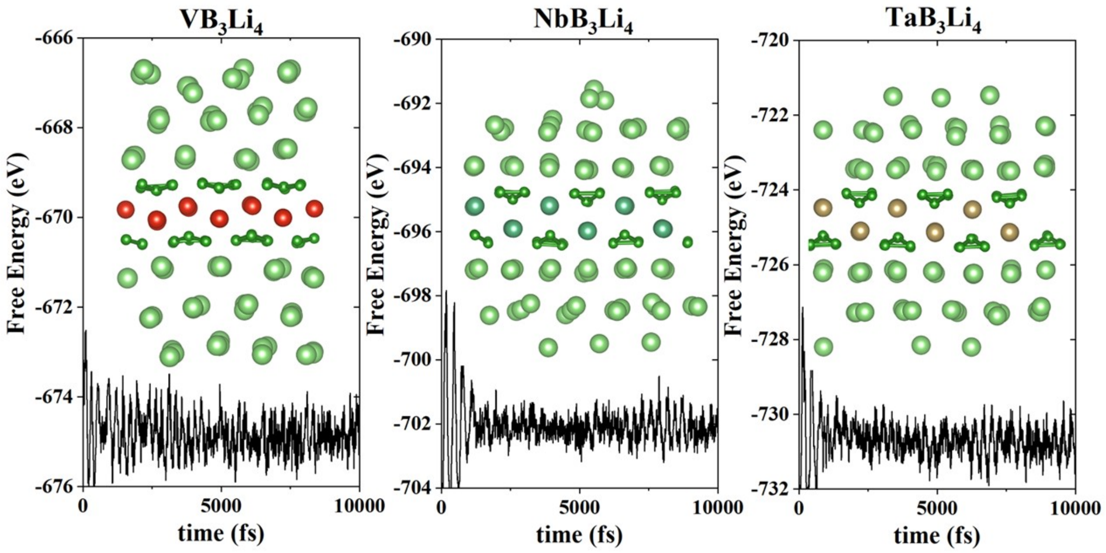

Fig. S3 The snapshots and energy curves of AIMD for VB_3Li_4, NbB_3Li_4 and TaB_3Li_4 monolayers at 300 K.
